# Expression of fatty acid synthase genes and their role in development and arboviral infection of *Aedes aegypti*

**DOI:** 10.1186/s13071-022-05336-1

**Published:** 2022-06-27

**Authors:** Nunya Chotiwan, Carlos A. Brito-Sierra, Gabriella Ramirez, Elena Lian, Jeffrey M. Grabowski, Babara Graham, Catherine A. Hill, Rushika Perera

**Affiliations:** 1 Department of Microbiology, Immunology and Pathology, Colorado State University, Fort Collins, CO USA; 2 Present Address: Chakri Naruebodindra Medical Institute, Faculty of Medicine Ramathibodi Hospital, Mahidol University, Samut Prakan, Thailand; 3 Department of Entomology, Purdue University, West Lafayette, IL USA; 4 Purdue Institute of Inflammation, Immunology and Infectious Disease, Purdue University, West Lafayette, IN USA; 5 Present Address: Foundation for Advanced Education in the Sciences at the NIH, Bethesda, MD USA; 6 Present Address: Lilly Research Laboratories, Eli Lilly and Company, IN Indianapolis, USA

**Keywords:** *Aedes aegypti*, Aag2 cells, Fatty acid synthase, FAS, Lipid, Lipid metabolism, Dengue virus, AaegL5 genome assembly

## Abstract

**Background:**

Fatty acids are the building blocks of complex lipids essential for living organisms. In mosquitoes, fatty acids are involved in cell membrane production, energy conservation and expenditure, innate immunity, development and reproduction. Fatty acids are synthesized by a multifunctional enzyme complex called fatty acid synthase (FAS). Several paralogues of FAS were found in the *Aedes aegypti* mosquito. However, the molecular characteristics and expression of some of these paralogues have not been investigated.

**Methods:**

Genome assemblies of *Ae. aegypti* were analyzed, and orthologues of human FAS was identified. Phylogenetic analysis and in silico molecular characterization were performed to identify the functional domains of the *Ae. aegypti* FAS (*Aa*FAS). Quantitative analysis and loss-of-function experiments were performed to determine the significance of different *Aa*FAS transcripts in various stages of development, expression following different diets and the impact of *Aa*FAS on dengue virus, serotype 2 (DENV2) infection and transmission.

**Results:**

We identified seven putative FAS genes in the *Ae. aegypti* genome assembly, based on nucleotide similarity to the FAS proteins (tBLASTn) of humans, other mosquitoes and invertebrates. Bioinformatics and molecular analyses suggested that only five of the *Aa*FAS genes produce mRNA and therefore represent complete gene models. Expression levels of *Aa*FAS varied among developmental stages and between male and female *Ae. aegypti*. Quantitative analyses revealed that expression of *Aa*FAS1, the putative orthologue of the human FAS, was highest in adult females. Transient knockdown (KD) of *Aa*FAS1 did not induce a complete compensation by other *Aa*FAS genes but limited DENV2 infection of Aag2 cells in culture and the midgut of the mosquito.

**Conclusion:**

*Aa*FAS1 is the predominant *Aa*FAS in adult mosquitoes. It has the highest amino acid similarity to human FAS and contains all enzymatic domains typical of human FAS. *Aa*FAS1 also facilitated DENV2 replication in both cell culture and in mosquito midguts. Our data suggest that *Aa*FAS1 may play a role in transmission of dengue viruses and could represent a target for intervention strategies.

**Graphical Abstract:**

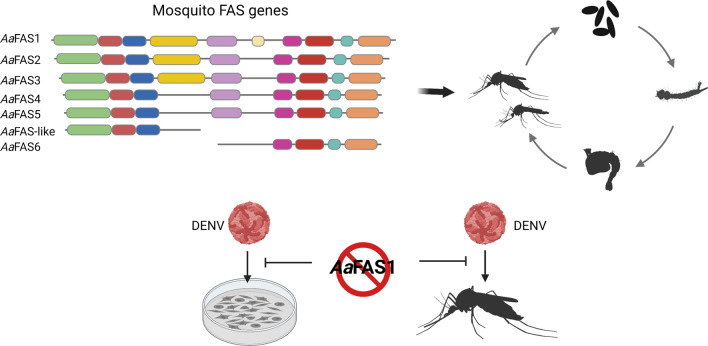

**Supplementary Information:**

The online version contains supplementary material available at 10.1186/s13071-022-05336-1.

## Background

Fatty acid synthase (FAS) is a multifunctional enzyme catalyzing > 40 steps in the de novo fatty acid biosynthesis pathway [1, 2]. It contains seven catalytic and three non-catalytic domains which condense, reduce and dehydrate the three-carbon substrate, malonyl-CoA, into 16–18-carbon fatty acids. These fatty acids are essential building blocks of complex lipids, such as phosphoglycerolipids, glycerolipids and sphingolipids, which are components of cellular membranes and storage lipids, and function as signaling molecules, respectively.

In mosquitoes, fatty acids also play roles in innate immunity, reproduction, development and flight [3–5]. Fatty acids can be acquired or synthesized in both larvae and adult stages. Neonate larvae acquire lipids through the maternal deposition in eggs [6–8] and through consumption of aquatic diets such as diatoms and algae, which are the primary source of polyunsaturated fatty acids [9]. Fatty acids from larval stages can be transferred to the adult stage and some can be deposited in eggs of the first gonotrophic cycle [10]. Adult mosquitoes possess enzymes for de novo synthesis and modification of fatty acids from both sugar (carbohydrate-enriched) and blood (protein-enriched) meals [7, 9, 11]. In the female, fatty acid synthesis is important for metabolism and production of eggs. Transient knockdown (KD) of acetyl-CoA carboxylase (ACC) and fatty acid synthase (FAS), two key enzymes in the de novo fatty acid biosynthesis pathway, led to significantly lower egg production in the first gonotrophic cycle [12]. In addition, eggs produced by ACC-KD mosquitoes lacked eggshells and were nonviable [12].

Apart from its importance to mosquito biology, studies suggest FAS also plays a supportive role for several arboviral infections in both mammalian and mosquito cells [13–16]. Several RNA viruses induce expansion and rearrangement of host cell membranes to support viral genome replication and assembly [17–19]. Studies have shown that FAS facilitates the production of dengue virus serotype 2 (DENV2) infection in both human and mosquito cells, potentially by providing the building blocks for this membrane expansion event [13, 15]. Lastly, studies also reported the elevation of fatty acid abundance in C6/36 (*Aedes albopictus*) cells and in the *Aedes aegypti* mosquito midgut during DENV2 infection [15, 20]. These findings suggest that fatty acids are essential for the physiological function of mosquitoes and support DENV2 infection of the mosquito.

Currently, understanding of FAS in mosquitoes and its role in pathogen transmission by the mosquito vector is limited. Here, we describe the molecular and functional characterization of the FAS gene family from *Ae. aegypti* (*Aa*FAS)*.* We identified seven putative *Aa*FAS genes (*Aa*FAS 1–6 and *Aa*FAS-like) in the AaegL5 genome assembly, characterized the expression of these genes during mosquito development and following consumption of different diets. *Aa*FAS1 has the highest amino acid similarity to human FAS and is the predominant transcript. We investigated the role of *Aa*FAS1 in DENV2 infection in mosquito cells and mosquito vectors using gene KD. We observed a significant reduction of DENV2 replication following *Aa*FAS1-KD in *Ae. aegypti* cells and a transient reduction of infection in *Ae. aegypti* midguts at early time points post-infectious blood meal. These results provide insights into the molecular characteristic of *Aa*FASs and their roles during *Ae. aegypti* development, food source acquisition and arbovirus infection.

## Methods

### Alignments, conserved motifs and phylogenetic tree

Putative *Aa*FAS sequences from the AaegL5 genome assembly were blasted against the AaegL3 genome assembly retrieved from VectorBase using tBLASTn [21–23]. FAS sequences of *Anopheles gambiae, Drosophila melanogaster, Apis mellifera, Homo sapiens*, *Mus musculus* and *Saccharomyces cerevisiae* were aligned with putative *Aa*FAS sequences using ClustalW [24]. mRNA sequences were retrieved from NCBI and manually curated to confirm the intron/exon boundaries. Conserved FAS motifs were identified by global alignment of vertebrate, invertebrate and yeast proteins using Clustal Omega [25] and Jalview 2.11.1.5 (accession numbers are shown in Additional file 1: Table S1) [26], and conserved amino acids associated with catalytic domains of functional FAS were identified by comparison to sequences reported in published studies [27]. Individual amino acid alignments were also performed between FAS-AaegL5 and FAS-AaegL3 using ClustalW to identify improvements in AaegL5 models.

A Bayesian inference of phylogeny was performed using the amino acid sequence of FAS from *Ae. aegypti*, *An. gambiae*, *D. melanogaster*, *A. mellifera*, *Mus musculus* and *H. sapiens*. Yeast Kexin was used as an outgroup. A sequence alignment with ClustalW was performed prior to tree construction in phylogeny.fr. The substitution model used for the Bayesian inference was Blosum62, and the Markov Chain Monte Carlo parameters included 100,000 generations with sampling every 10 generations, discarding the first 250 trees. The resulting tree was annotated and curated in iTOL [28].

### Annotation of protein domains in *Aa*FAS genes

*Aa*FAS amino acid sequences were aligned against the human FAS (NP_004095.4, NCBI) using Clustal Omega to identify the seven catalytic and three noncatalytic domains associated with mammalian FAS. The alignment results were viewed using MView tool [29]. Motifs in the human FAS were identified based on Pfam 31.0 [30], and conserved domains in *Aa*FAS genes were identified by comparative analyses.

### Mosquito rearing

Larvae of *Ae. aegypti* strain Chetumal, originally collected from Yucatan Peninsula in Mexico, were reared on fish food. Adult mosquitoes were reared on 10% sucrose solution and maintained under constant conditions of 28 °C, 80% relative humidity [31].

### Blood feeding

Mosquitoes were starved by removal of sucrose solution and water for 24 and 4 h, respectively, prior to blood feeding. Defibrinated sheep blood (Colorado Veterinarian Product) was mixed with 1 mM ATP and placed in an artificial membrane feeder warmed by a 37 °C water jacket. Mosquitoes were allowed to feed for 45–60 min. Only fully engorged mosquitoes were used for the experiment and were reared on 10% sucrose solution and water.

### Generating long double-stranded RNA

Long double-stranded RNA (dsRNA) was generated from adult female *Ae. aegypti* total RNA. Primers were designed to amplify an  ~ 500-bp region of the gene of interest (Additional file 1: Table S2). cDNA was generated by reverse transcription (RT) using specific reverse primers and SuperScript III Reverse Transcriptase (Invitrogen). Polymerase chain reaction (PCR) was performed using specific primers containing a 5′ T7 promotor sequence adapted to both forward and reverse primers and Taq polymerase (NEB). PCR products were purified using the GeneJET PCR Purification kit (Thermo Scientific), and in vitro transcription was performed using the MEGAscript T7 kit (Invitrogen) and incubation at 37 °C for 12 h. Following incubation, the product was heated to 75 °C for 5 min and slowly cooled to room temperature for 4 h to dsRNA annealing. Next, dsRNA was treated with DNase (NEB) and purified by phenol–chloroform extraction followed by ethanol precipitation and the purified dsRNA was stored at − 80 °C.

### dsRNA knockdown of *Aa*FAS1 in *Ae. aegypti*

dsRNA was introduced via intrathoracic (IT) injection of adult females at 3–4 days post-eclosion [32]. Mosquitoes were anesthetized at 4 °C on a cold plate. Glass needles were prepared with a vertical pipette puller (P-30, Sutter Instrument Co., Novato, CA), and mosquitoes were IT injected with 3 µg/µl of dsRNA in an injection volume of 69 nl twice (total of ~ 400 ng of dsRNA) using a Nanojet II (Drummond Scientific Company, Broomall, PA). Injected mosquitoes were fed on sucrose solution or blood and reared at 28 °C, 80% relative humidity for 17 days post-injection.

### dsRNA knockdown of *Aa*FAS1 gene and DENV2 infection of Aag2 cells

dsRNA KD was performed in RNA interference-competent *Ae. aegypti* (Aag2) cells. Aag2 cells were cultured in Schneider’s insect medium (Sigma-Aldrich) supplemented with 2 mM L-glutamine, 1% non-essential amino acids and 10% fetal bovine serum (FBS). The cells were seeded in a 48-well plate at 50,000 cells/well for 24 h and subsequently transfected with 260 ng of dsRNA, against *Aa*FAS1, DENV2 (positive KD control) or GFP (negative KD control) genes, mixed with TransIT-2020 Reagent (Mirus) following the manufacturer’s protocol. New medium with 2% FBS was replaced at 6 h post-transfection. Cell viability assays were performed at 2 days post-transfection using resazurin assay.

KD cells were infected with infectious DENV2 expressing a luciferase reporter (DEN-Luc) supplied by C. Rice, Rockefeller University. Cell culture medium was replaced with 300 µl of DEN-Luc supernatant at 48 h post dsRNA transfection, and cells were incubated at 28 °C without CO_2_. Virus supernatant was removed at 24 h post-infection, and cells were lysed. Luciferase activity was read using the Luciferase Assay System (Promega) as per manufacturer protocol.

### Gene expression analyses

Total RNA was extracted from dissected midgut or whole mosquito by TRIzol (Life Tech), and cDNA was produced via reverse transcription using random primers (Life Tech) and SuperScript III Reverse Transcriptase (Invitrogen). Approximately 400 ng of total cDNA was employed for quantitative PCR (qPCR) analyses. Gene-specific primers are listed in Additional file 1: Table S3. β-Actin was used as a reference gene. Relative *Aa*FAS gene expression was assessed by normalization to the levels of the β-actin gene (2^−ΔCt^). The comparative Ct (2^−ΔΔCt^) method was used to calculate the relative expression of *Aa*FAS following treatment compared to the control [33].

For RT-PCR assay, total RNA was treated with DNase I, RNase-free (1 U/µl) kit (ThermoFisher) prior to reverse transcription reaction. Purified cDNA was then amplified using Q5^®^ High-Fidelity DNA Polymerase kit (New England BioLabs) with following conditions: 98 °C for 30 s, 35 cycles of 98 °C for 10 s, 68 °C and 72 °C for 2 min and 30 s. Primers are listed in Additional file 1: Table S4.

### Virus infection of *Ae. aegypti* by infectious blood meal

DENV2 serotype 2 strain Jamaica-1409 [34] was cultured in C6/36 cells. Cells were infected with DENV2 at a multiplicity of infection of 0.01 and incubated at room temperature for 1 h. Virus supernatant was removed, and infected cells were cultured in 5 ml total volume of L15 medium supplemented with 3% FBS, 50 μg/ml penicillin–streptomycin and 2 mM L-glutamine. Media were replaced at 7 days post-infection (dpi), and virus supernatant was harvested at 12–14 dpi and immediately used for infectious blood feeding.

To prepare the infectious blood meal, infected cells were scraped from the bottom of the cell culture flask using a cell scraper. A mixture of cells and virus supernatant was added to the defibrinated sheep blood at 1:1 ratio and supplemented with 1 mM ATP. Female mosquitoes (3–7 days post eclosion) were prepared, fed with the infectious blood meal, sorted and reared as mentioned in the blood-feeding section above.

### Midgut dissection and plaque titration

Mosquito tissues were collected at multiple days post-exposure to the virus as indicated in the figure legends. Isolated midguts or the mosquito carcass (remainder of the body without midgut) were placed separately into 2-ml safe-lock Eppendorf tubes (Eppendorf) containing 250 µl of mosquito diluent [1 × PBS supplemented with 20% FBS, 50 µg/ml penicillin/streptomycin (Gibco), 50 µg/ml Gentamycin (Gibco) and 2.5 µg/ml Amphotericin B (Gibco)] and a stainless-steel bead [35]. Tissue was homogenized using a Retsch Mixer Mill MM400 at 24 cycles per second for 1 min and centrifuged at 15,000*g* for 5 min at 4 °C, and supernatant was transferred to a new tube for plaque titration.

Plaque assay was performed on BHK-15 cells. Ten-fold serially diluted viral supernatant was absorbed on the confluent cell layer. After 45 min of absorption, cells were overlaid with 1 × Minimum Essential Media (MEM), 1 × agar supplemented with 2.5% FBS, 25 µg/ml penicillin/streptomycin, 25 µg/ml gentamycin and 1.25 µg/ml amphotericin B, and the cells were incubated at 37 °C with 5% CO_2_. Cells were stained with 0.033% neutral red (Sigma) in 1 × PBS on day 5 post-infection, and plaques were counted at 24 h post-staining.

### Statistical analyses

Statistical analyses comparing gene expression or DENV2 viral load were performed by one-way ANOVA followed by Tukey’s multiple comparison tests. Values of gene expression or virus titer were reported as mean ± SD. Proportions of infection among groups on each day were compared using pairwise *χ*^2^ tests with *p*-values adjusted using a Holm correction for multiple comparisons [36], *p < 0.05, **p < 0.01, ***p < 0.005 and ****p < 0.001.

## Results

### Molecular characterization of *Aa*FAS genes

**Fig. 1:**
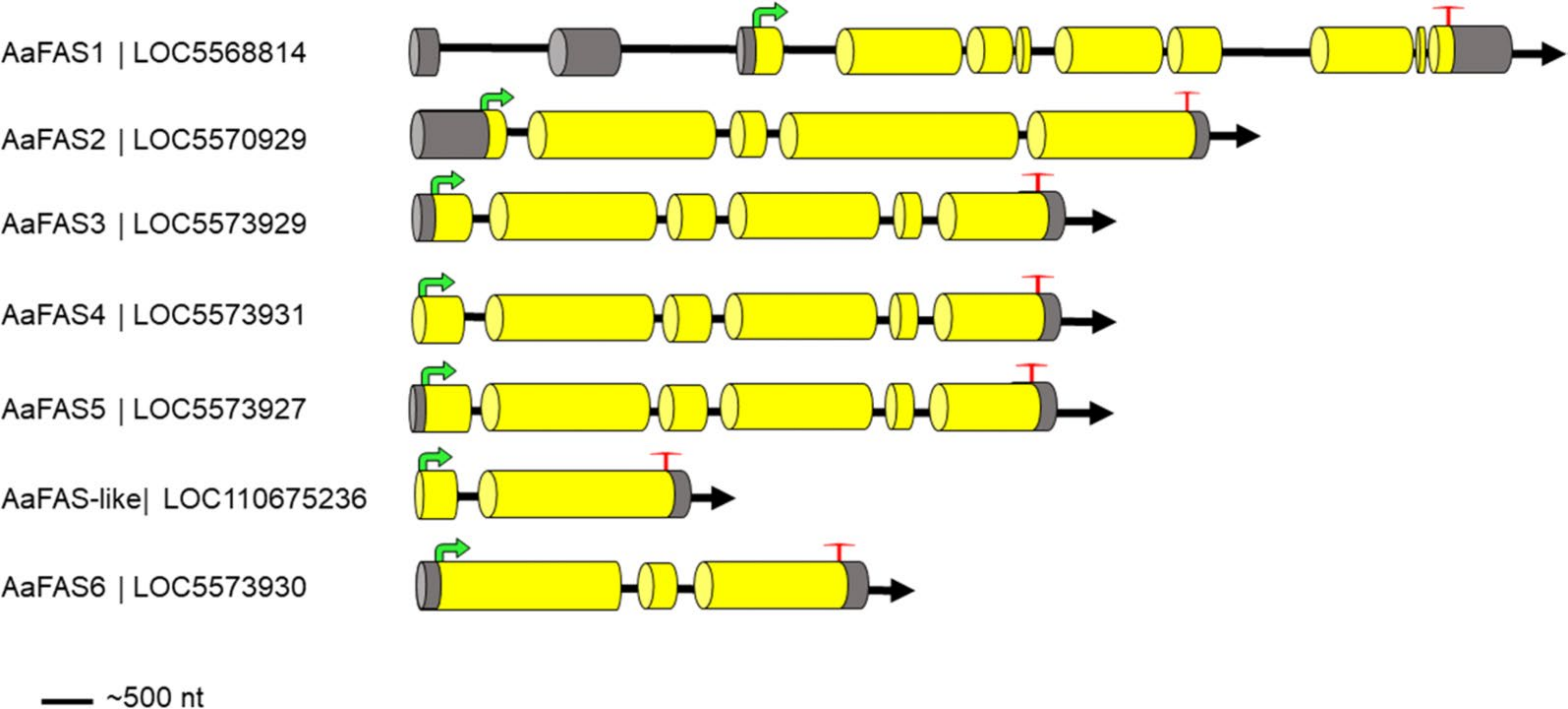
Schematic showing the predicted gene structure of the *Aa*FAS gene family. Exons are indicated by yellow cylindrical bars, 5′ and 3′ non-coding exons by dark gray shading, introns by a black line, start codon by green arrow and stop codon by red T

We obtained seven putative *Aa*FAS gene models via manual annotation using the AaegL5 assembly [37]. Previously, five candidate FAS genes (*Aa*FAS1-5) were identified based on the AaegL3 assembly of Nene et al., 2007 [23, 38], and of these, only *Aa*FAS1 and 2 underwent functional studies [12]. The AaegL5 assembly enabled identification of two additional candidate FAS genes (*Aa*FAS6 and *Aa*FAS-like). The corresponding mRNA sequences showing predicted intron/exon structure, and initiation and stop codons are shown in Additional file 2. The *Aa*FAS1 gene model revealed a gene structure comprising 11 exons, while *Aa*FAS2 had 5 exons and *Aa*FAS3*-*5 had 6 exons (Fig. 1). The incomplete *Aa*FAS-like and *Aa*FAS6 gene models comprised 2 and 3 exons, respectively.

The gene models for *Aa*FAS1-5 appeared to be full length, with an average gene product length of 2360 amino acids (Table 1). *Aa*FAS1-5 possessed features associated with functional FAS, including an initiation methionine, a stop codon and the functional catalytic motifs (DTACSS, EAH and GSVKS) important for ketoacyl synthesis as described by Beedessee et al. [27]. Additionally, *Aa*FAS1-5 containing the YKELRLRGY motif conserved among the FAS genes of vertebrates and invertebrates presents in the polyketide synthase dehydratase domain (Additional file 1: Fig. S1). *Aa*FAS3 lacked six amino acid residues in the 3′ terminus of exon 6. We also identified a total of 127 non-synonymous substitutions in this model compared to its AaegL3 counterpart.

**Table 1:** Summary of *Aa*FAS gene family predicted from the *Aedes aegypti* AaegL5 assembly

Name	NCBI accession number (AaegL5)	VectorBase accession number (AaegL3)	Max. number of exons	Chro-mo-some	Location	% Ident with human FAS	Length (nucleotides)	Length (amino acids)	No. splice variants/isoforms	Notes on the revised annotation	% identity (AaegL3 and L5)
*Aa*FAS1	LOC5568814	AAEL001194	11	2	NC_035108.1 (307544012..307601765, complement)	45.3	9732	2422	1	1 SNP in exon_4	99.9
*Aa*FAS2	LOC5570229	AAEL008160	5	3	NC_035109.1 (9993811..10002427, complement)	36.7	8368	2385	1	1 SNP in exon_5	100
*Aa*FAS3	LOC5573929	AAEL022506	6	2	NC_035108.1 (429256663..429264110, complement)	32.9	7144	2334	1	18 SNPs exon_1, 31 SNPs exon_2, 11 SNPs exon_3, 52 SNPs exon_4, 3 SNPs exon_5, and 12 SNPs exon_6. Deletion of six amino acid residues when compared with the AaegL3 orthologue	94.6
*Aa*FAS4	LOC5573931	AAEL002237	6	2	NC_035108.1 (429327062..429334421, complement)	34.6	7068	2333	1	4 SNPs exon_2, 10 SNPs exon_4, 6 SNPs exon_6	99.1
*Aa*FAS5	LOC5573927	AAEL002228	6	2	NC_035108.1 (429228612..429236010, complement)	32.9	7097	2324	1	5 SNPs exon_2, 2 SNPs exon_3, 3 SNPs exon_4, 1 SNP exon_6	99.5
*Aa*FAS-like	LOC110675236	–	2	2	NC_035108.1 (429280401..429282870, complement)	36.1	2409	800	1	–	–
*Aa*FAS6	LOC5573930	–	3	2	NC_035108.1 (429275401..429279876, complement)	36.8	4353	1386	1	–	–

The AaegL5 annotation is shown in comparison to the AaegL3 gene models reported by Nene et al. [23]

**Fig. 2:**
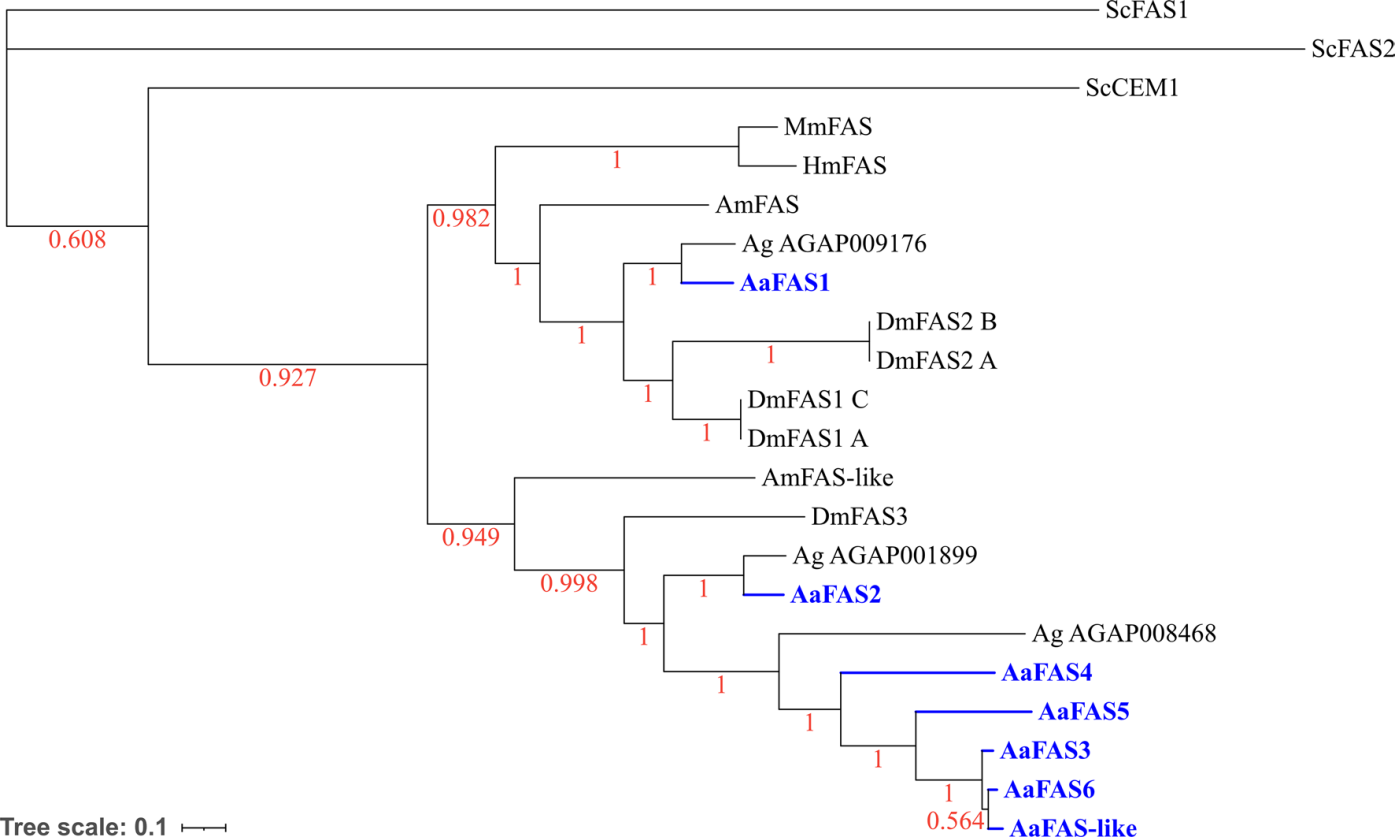
Phylogenetic analysis of *Aa*FAS. Bayesian phylogeny represented with an unrooted tree showing the main relationships between mosquito FAS genes and their counterparts in Ag: *Anopheles gambiae,* Dm: *Drosophila melanogaster,* Am: *Apis mellifera,* Mm: *Mus musculus*, Hm: *Homo sapiens* and Sc: *Saccharomyces cerevisiae*. The branches are supported by posterior probability values. The tree is drawn to scale: amino acid changes per site

The Bayesian inference supported *Aa*FAS1-5 as paralogues and revealed highest percent amino acid similarity between *Aa*FAS1 and *H. sapiens* FAS (human FAS) (Fig. 2). Notably, *Aa*FAS1 clustered in a clade comprising the *H. sapiens*, *Mus musculus*, *A. mellifera* FAS, *D. melanogaster* FAS1 and 2, and an uncharacterized *An. gambiae* FAS (Fig. 2). Similarly, *Aa*FAS2 clustered in a clade with another uncharacterized *An. gambiae* FAS. In contrast, *Aa*FAS3, 4, 5, 6 and -like clustered at the most branched portion of the tree, suggesting a recent diversification event. Phylogenetic analyses and amino acid alignment supported *Aa*FAS1-5 as the counterparts of the AaegL3 genome assembly-derived gene models as follows: LOC5568814-AAEL001194; LOC5570229-AAEL008160; LOC5573929-AAEL022506; LOC5573931-AAEL002237 and LOC5573927-AAEL002228 (Fig. 2, Table 1). *Aa*FAS-like and *Aa*FAS6 (LOC110675236 and LOC5573930) were not identified in the AaegL3 assembly, suggesting these models are unique to the AaegL5 assembly.

**Fig. 3:**
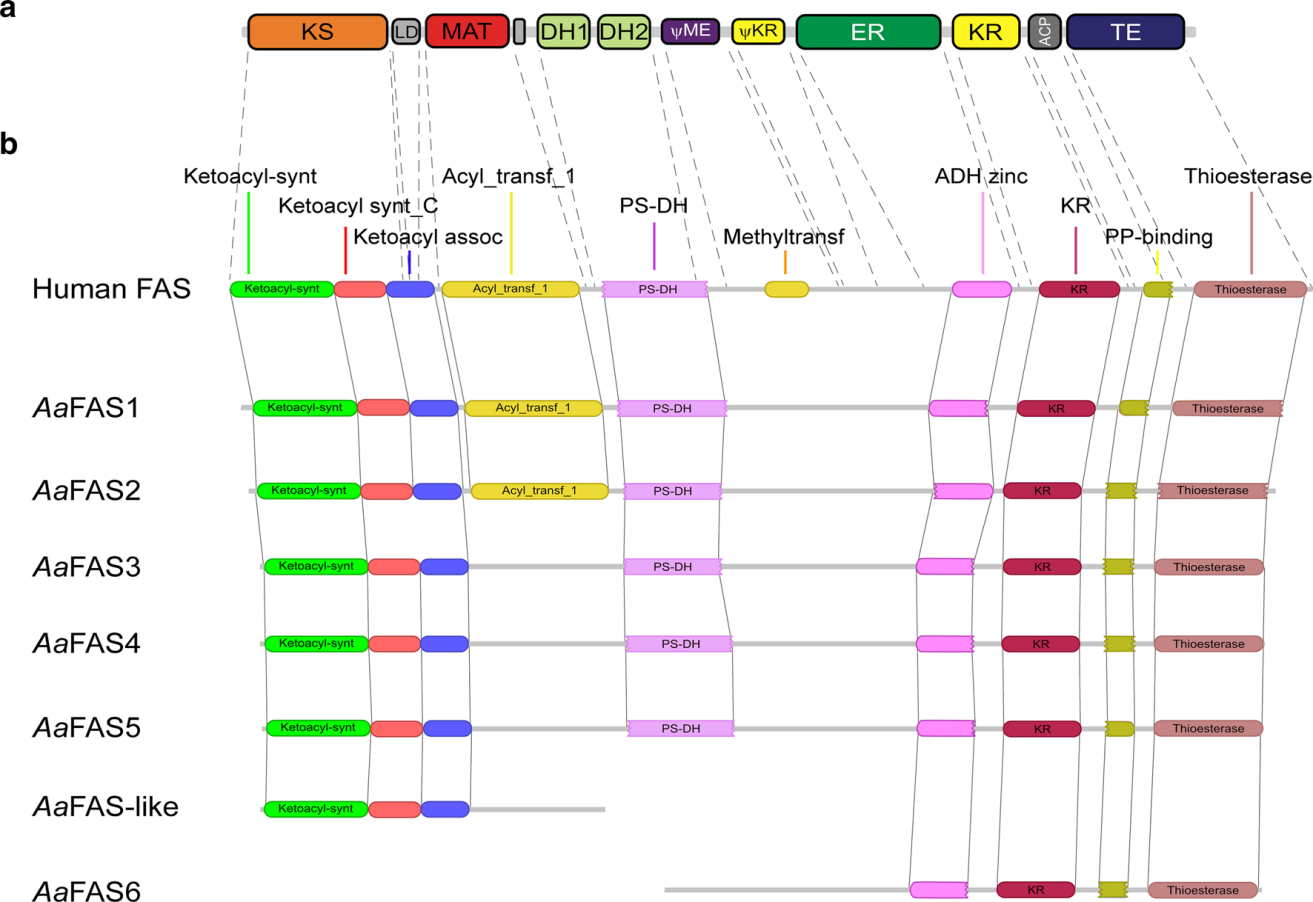
Linear organization of *Aedes aegypti* FAS genes showing functional domains. (**a**) Schematic shows linear organization of seven catalytic and three noncatalytic domains of mammalian FAS annotated by Maier et al. [1]. Seven catalytic domains are shown in big squares, and three non-catalytic domains are shown in smaller squares. *KS* β-ketoacyl synthase; *LD* linker; *MAT* malonyl-acetyl transferase; *DH* dehydratase; *ΨME* pseudo-methyltransferase; *ΨKR* pseudo β-ketoacyl synthase; *ER* enoyl reductase; *KR* β-ketoacyl synthase; *ACP* acyl carrier protein and *TE* thioesterase. **b** Schematics show conserved domains or motifs of FAS genes and their organization annotated using Pfam 31.0 software. *ketoacyl_synt* β-ketoacyl synthase; *ketoacyl_synt_C* β-ketoacyl-acyl carrier protein synthase; *ketoacyl_assoc* ketoacyl-synthase C-terminal extension; *acyl_transf_1* acyl transferase domain; *PS-DH* polyketide synthase; *methyltransf* methyltransferase domain; *ADH-zinc* zinc binding dehydrogenase; *KR* β-ketoreductase domain; *PP-binding* phosphopantetheine attachment site; *thioesterase* thioesterase domain

To investigate putative functional domains, we aligned *Aa*FAS sequences to the human FAS using Clustal Omega [25]. Human FAS contains seven catalytic domains and three noncatalytic domains [1]. Collectively, *Aa*FAS genes possessed < 50% amino acid identity to human FAS, and of the seven gene models, *Aa*FAS1 had the highest amino acid identity (45.3%) (Table 1 and Additional file 1: Table S5). Alignment of FAS domains also showed modest sequence identity between human FAS and *Aa*FAS (23.03–63.56%) with greatest similarity for *Aa*FAS1 domains (Additional file 1: Table S5). The linear organization of mammalian FAS domains annotated by Maier et al. is shown in Fig. 3 [1]. Using Pfam 31.0 software, we identified the conservation in linear organization of motifs associated with known functional domains (Fig. 3B). Dotted lines indicate motif boundaries between mammalian FAS domains (Fig. 3A) and *Aa*FAS domains (Fig. 3B). Pfam analysis did not show the presence of functional methyltransferase domains in *Aa*FAS (Fig. 3B), and protein sequence alignment using Clustal Omega showed deletion within pseudo-methyltransferase (ΨME) domains of *Aa*FAS compared to human FAS (16.20–23.03% identity; Additional file 1: Table S5 and Fig. S2).

The gene model of *Aa*FAS-like was 800 amino acids in length and contained all functional catalytic motifs, whereas *Aa*FAS6 was 1386 amino acids in length and lacked catalytic motifs but contained the conserved 3′ motif YKELRLRGY conserved in FAS (Additional file 1: Fig. S1). *Aa*FAS-like contains ketoacyl synthase, ketoacyl synthase_C and ketoacyl-synthase C-terminal extension domains, the first 5′ domains of *Aa*FAS1-5 and human FAS (Fig. 3B), while *Aa*FAS6 contains ADH zinc, β-ketoreductase, PP binding and thioesterase domains, the last four domains located 3′ in *Aa*FAS1-5 and human FAS (Fig. 3B). In the AaegL5 assembly, *Aa*FAS-like and 6 locate on chromosome 2 at positions 429280401-429282870 and 429275401-429279876, respectively. It is possible that *Aa*FAS-like and -6 reflect an error in genome assembly or a gene duplication. However, the molecular data and the inability to detect transcripts associated with either locus (Additional file 1: Fig. S3) suggest that *Aa*FAS-like and 6 represent pseudogenes (Fig. 3B).

### FAS expression during *Ae. aegypti* development

Mosquitoes undergo four developmental stages: egg, larva, pupa and adult. We used RT-qPCR to explore the hypothesis that expression patterns of *Aa*FAS genes vary among these stages. We collected five individual mosquitoes for each of the fourth larval instar, pupa and adult stages.

**Fig. 4:**
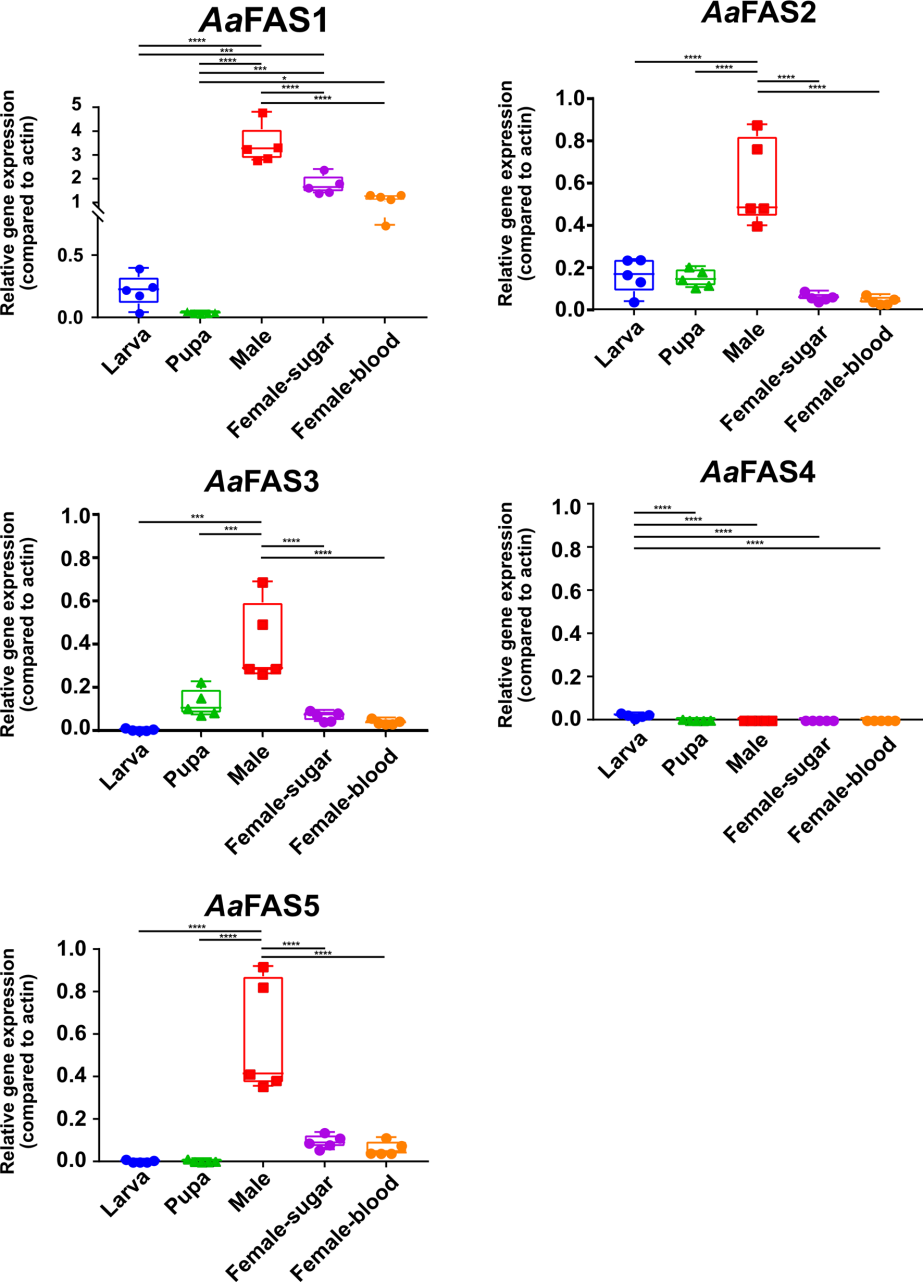
Expression of *Aa*FAS in mosquito developmental stages and sexes. RNA was prepared from five specimens of fourth-instar larvae, pupae, sugar-fed males, sugar-fed females and blood-fed females (3-days pbm) (all adult mosquitoes were collected at the same day time; 8–10 days post eclosion). Samples were subjected to RT-qPCR to assess relative expression of *Aa*FAS1-5 and *Aa*FAS-like. RNA levels between samples were normalized to the β-actin gene using 2^−ΔCt^ method. The boxes show the 25th and 75th percentiles, the whiskers show the minimum and maximum values. The midline indicates the median of the relative gene expression value. The experiment was repeated twice, and representative results from a single experiment are shown

Relative expression analyses revealed negligible *Aa*FAS expression in larval and pupal stages, while we observed the highest expression of all genes except *Aa*FAS4 in adult males (Fig. 4). *Aa*FAS1 was the most predominant FAS expressed in adult mosquitoes. We did not observed differences in expression levels of any *Aa*FAS genes between sugar-fed and 3 days post blood-fed (coinciding with the first gonotrophic cycle) females. The study also revealed negligible *Aa*FAS4 expression in all developmental stages and sexes (Fig. 4).

### Impact of blood feeding on expression of *Aa*FAS1

**Fig. 5:**
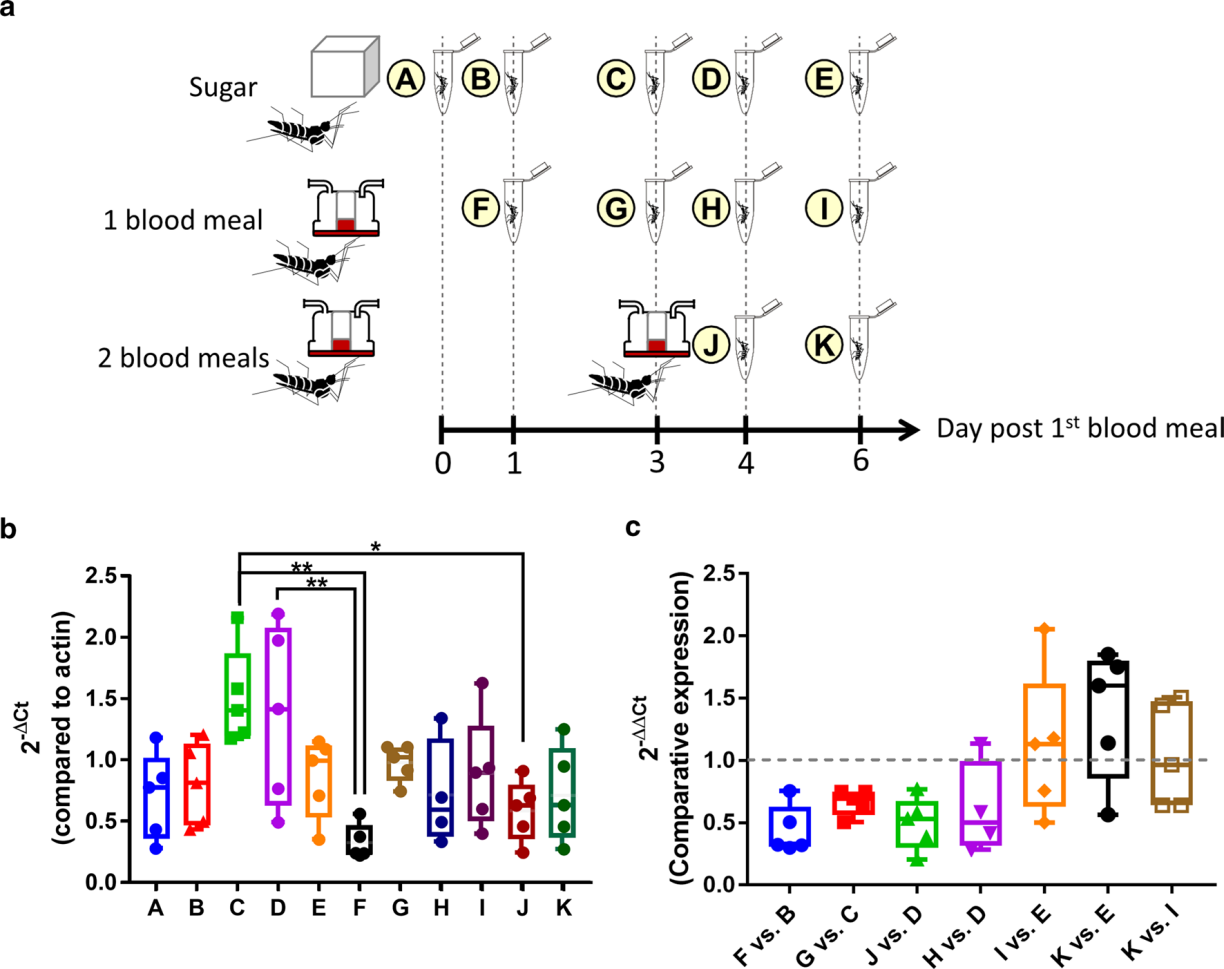
Expression of *Aa*FAS1 in sugar-fed and blood-fed mosquitoes. **a** Schematic of the experimental design. Mosquitoes were reared in three different conditions: sugar-fed only, one blood meal, which were fed on day zero, and two blood meals, which were again fed on day 3. Mosquitoes in all conditions were also allowed access to additional sugar and water all the time throughout the experiment. Five mosquitoes from each condition were collected on different days post first blood meal feeding designated in letters A–K. **b** *Aa*FAS1 expression profile (2^−ΔCt^; normalized to actin) of each sampling group were shown. **c** Expression of *Aa*FAS1 was measured by the comparative expression (2^−ΔΔCt^) method. The samples used for comparisons are shown on the X-axis. Boxes show the 25th and 75th percentiles, whiskers show the minimum and maximum values, and the midline shows the median of the relative gene expression value

The diet of the female *Ae. aegypti* typically involves both nectar and blood. The blood meal is rich in proteins and lipids; therefore, this diet may trigger lipolysis, instead of synthesis, to break down lipid molecules. We compared *Aa*FAS1 expression, the predominant *Aa*FAS in adult females, in sugar-fed females versus blood-fed females (feeding once or twice) (Fig. 5). Blood meals were provided only on specific days as shown in Fig. 5A, while mosquitoes from all groups were fed ad lib on 10% sugar diet throughout the experiment. Comparisons of *Aa*FAS1 gene expression from mosquito samples collected on the same day showed no differences among feeding conditions (Fig. 5B). However, when profiled as ratios (Fig. 5C), we observed a slight, but not significant, reduction of *Aa*FAS1 expression in females given a single blood-meal compared to sugar-fed females on days 1, 3 and 4 post-blood meal (pbm) (Fig. 5B: F vs. B, G vs. C and H vs. D). These data suggest that diet may only play a minor role, if any, in the expression of *Aa*FAS1 gene.

### Transient knockdown of *Aa*FAS1 gene causing upregulation of other *Aa*FAS genes

**Fig. 6:**
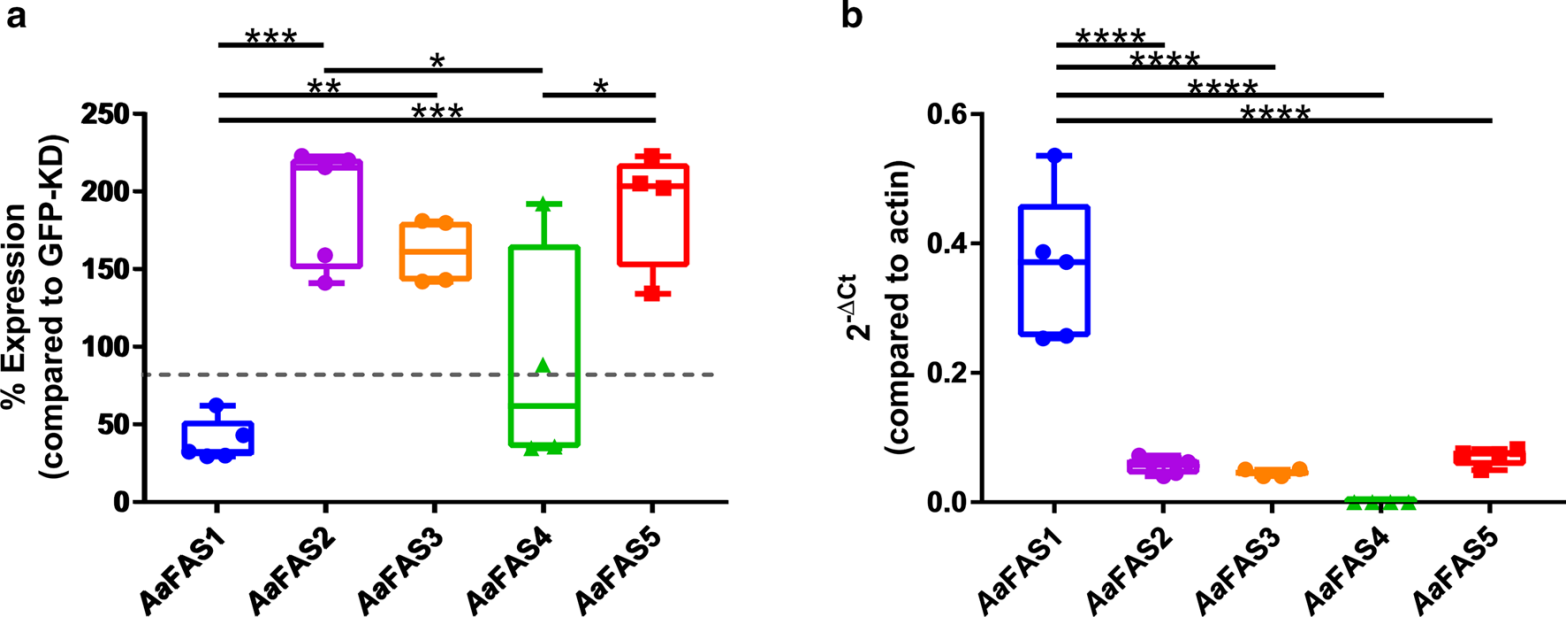
Comparative analyses of *Aa*FAS expression in *Aa*FAS1-KD mosquito. Three-day-old adult female mosquitoes were intrathoracically injected with dsRNA derived from *Aa*FAS1 or GFP mRNA sequence (an irrelevant dsRNA control). Mosquitoes were reared on 10% sugar diet for 2 days post-injection. Five mosquitoes were collected for *Aa*FAS gene expression measurements. **a** Percent relative expression of *Aa*FAS genes in *Aa*FAS1-KD mosquitoes was compared to GFP-KD control mosquitoes using the comparative Ct (2^−ΔΔCt^) method. **b** Gene expression profiles of *Aa*FAS were measured as normalized to β-actin gene expression (2^−ΔCt^). Boxes show the 25th and 75th percentiles, whiskers show the minimum and maximum values and midline shows median of the relative gene expression value. The experiment was repeated twice, and representative results from a single experiment are shown

We hypothesized that the redundancy of *Aa*FAS genes may serve as a backup system for the mosquitoes. To test this hypothesis, we employed *Aa*FAS1 loss-of-function studies to investigate the possibility of compensation by other *Aa*FAS genes. Female mosquitoes were IT injected with dsRNA derived from *Aa*FAS1 or GFP (KD control). On day 2 post-dsRNA injection, five mosquitoes were collected for assessment of *Aa*FAS expression (Fig. 6). We observed an approximate 40% reduction in *Aa*FAS1 expression (~ 39.3 ± 13.9%) in *Aa*FAS1-KD mosquitoes compared to the GFP-KD control (Fig. 6A). In *Aa*FAS1-KD mosquitoes, expression levels of *Aa*FAS2, 3 and 5 were 191.7 ± 38.6%, 161.4 ± 21.8% and 191.1 ± 38.9%, respectively, compared to their levels in GFP-KD control, indicating possible compensation for the loss of *Aa*FAS1 transcript. Conversely, the expression of *Aa*FAS4 was 87.71 ± 74.0% compared to *Aa*FAS4 expression in GFP-KD control mosquitoes. To determine whether the upregulation observed in *Aa*FAS2, 3 and 5 could possibly compensate for the loss of *Aa*FAS1 in the *Aa*FAS1-KD mosquitoes, we normalized the level of *Aa*FAS genes to β-actin. We observed modest expression of *Aa*FAS transcripts (5.6 ± 1.44% for *Aa*FAS2, 4.6 ± 0.00% for *Aa*FAS3 and 7.07 ± 0.62% for *Aa*FAS5 compared to β-actin), while these upregulations still did not match the remnant of *Aa*FAS1 expression after the KD effect (36.1 ± 11.6%). These data suggest that other *Aa*FASs may not be able to serve as a backup system for *Aa*FAS1, at least in adult female mosquitoes under transient KD condition.

### Effect of RNAi-induced *Aa*FAS1 knockdown on DENV2 replication in *Ae. aegypti* cells

**Fig. 7:**
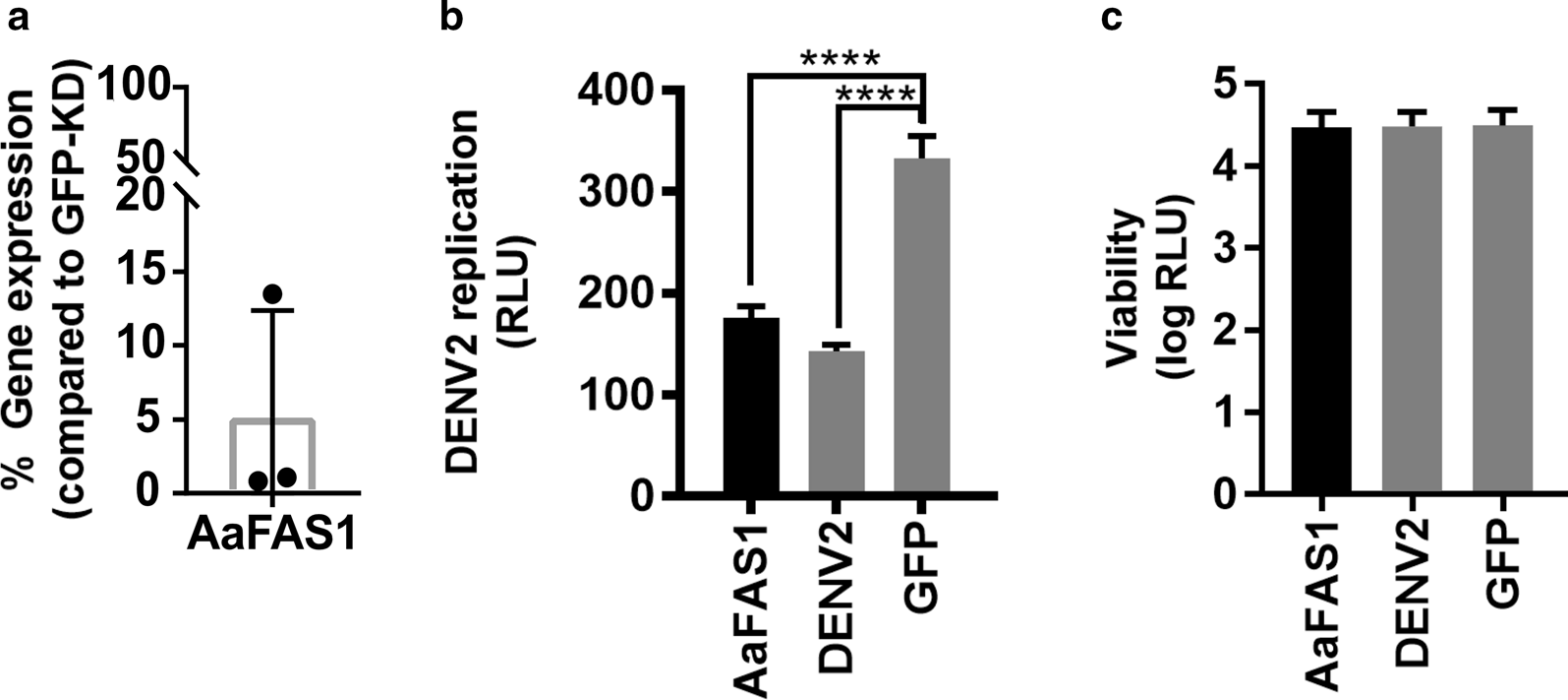
Assessment of RNAi-induced *Aa*FAS1 knockdown on DENV2 replication in Aag2 cells. **a** Percent *Aa*FAS1 expression in *Aa*FAS1-KD Aag2 cells compared to the *Aa*FAS1 expression in GFP-KD cells. Expression was measured at 2 days post dsRNA transfection. **b** RNA replication of luciferase-expressing DENV2 in Aag2 cells treated with dsRNA derived from *Aa*FAS1, DENV2 (positive KD control) or GFP (negative KD control). Cells were transfected with dsRNA for 2 days prior to infection with luciferase-tagged DENV2. At 24 h post infection, cells were lysed and were assayed for luciferase activity (RLU). **c** Viability of Aag2 cells treated with dsRNA derived from *Aa*FAS1, DENV2 and GFP genes assessed by resazurin assay. All treatment were performed on three biological replicates. The experiment was repeated twice, and representative results from a single experiment are shown

Studies in cell culture have shown that FAS activity is required for flavivirus genome replication [13, 14, 39]. Biochemical inhibition of FAS activity reduced DENV2 replication in both human and mosquito C6/36 cells [13, 15, 16]. The lack of functional RNAi machinery in C6/36 cells hindered the use of transient KD strategy in mosquito cells. However, *Ae*. *aegypti* cells, Aag2, have functional RNAi machinery; therefore, we can investigate the role of *Aa*FAS1, the most abundant transcript in female mosquitoes, in DENV2 replication using dsRNA transient KD in these cells [40]. At 48 h post-*Aa*FAS1-KD (time zero of DENV2 infection), the expression level of *Aa*FAS1 in Aag2 cells was 5.13 ± 7.24% compared to *Aa*FAS1 expression in GFP-KD negative KD control cells (Fig. 7A). At 24 h post DENV2 infection (72 h post-KD), we observed significant reduction (*P* < 0.001) in DENV2 RNA replication in *Aa*FAS1-KD cells compared to the GFP-KD controls, comparable to replication in DENV2-KD (positive KD control) (Fig. 7B), while the KD was not associated with detrimental effects to the cells (Fig. 7C), suggesting that *Aa*FAS1 is required for DENV2 replication in mosquito cells.

### Transient inhibition of *Aa*FAS1 reduced DENV2 infection in the midgut of *Ae. aegypti*

**Fig. 8:**
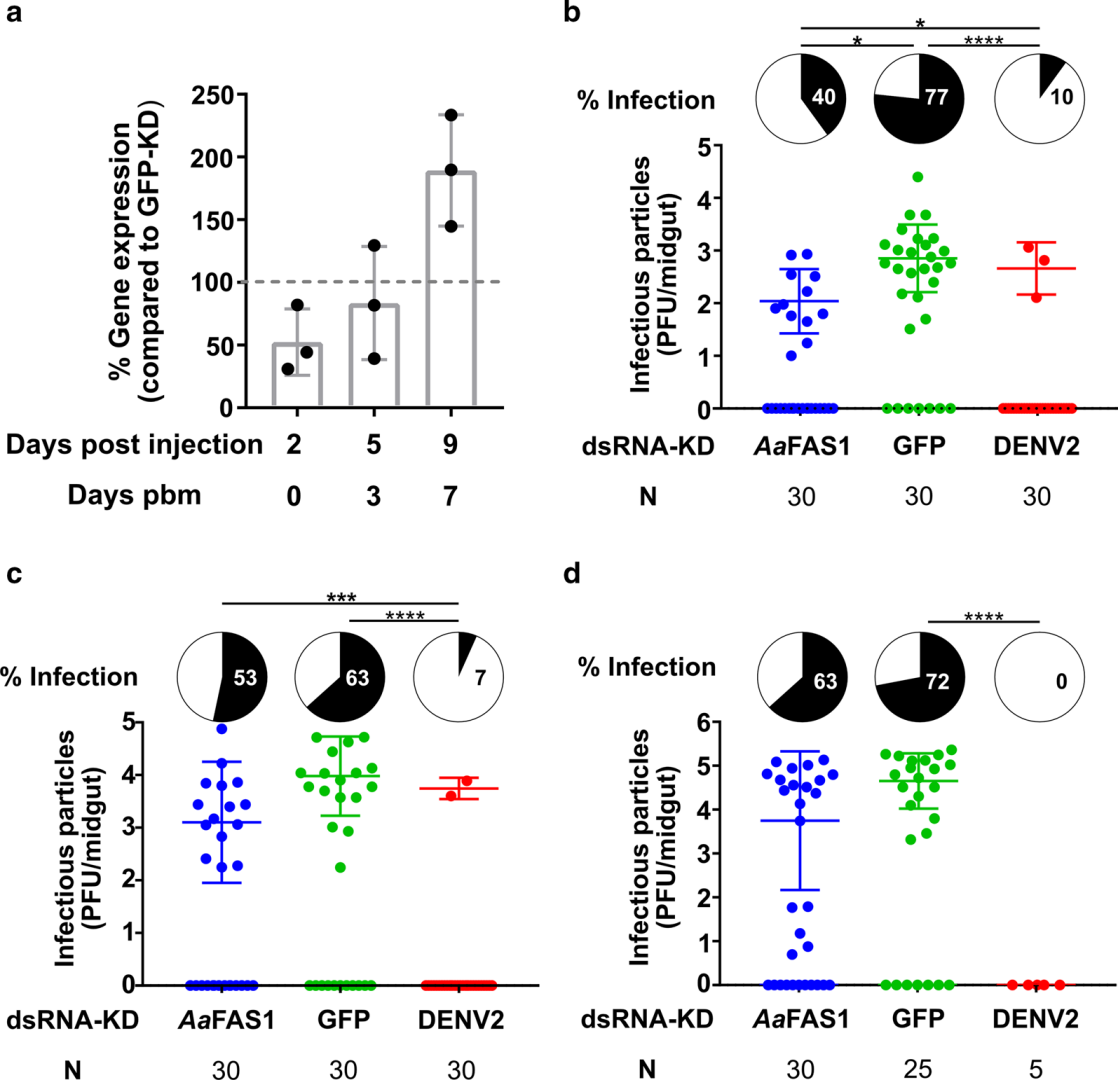
Transient KD of *Aa*FAS1 expression by dsRNA temporarily reduced DENV2 infection in midguts. **a** Percent *Aa*FAS1 expression in *Aa*FAS1-KD compared to GFP-KD mosquitoes. Mosquitoes were IT injected with  ~ 400 ng of dsRNA derived from *Aa*FAS1 or GFP (negative KD control) and fed with a blood meal 2 days post IT injection. On days 2, 5 and 9 post IT injection (days 0, 3 and 7 pbm), 3 pools of 5 mosquitoes from both treatments were collected and analyzed for *Aa*FAS1 expression. **b**–**d** Mosquitoes were IT injected with dsRNAs against *Aa*FAS1, GFP and DENV2 (positive KD control) and infected with DENV2 via infectious blood meal at 2 days post injection. Plaque assay was performed on midguts dissected on (**b**) day 3 and (**c**) day 7 and (**d**) carcasses (whole body without midgut) collected on day 14 pbm. Pie charts (black) show percent infected mosquito tissue in each treatment. Pairwise *χ*^2^ tests with Holm’s correction for multiple comparisons were used to analyze differences in proportion of infected mosquitoes among groups. Dot plots report virus titer in mosquito tissues. Mean virus titer (infectious particles) was calculated for infected samples only. (i) and (ii) indicate the separation of DENV2 titers in the carcass (day 14) that were produced from the *Aa*FAS1-KD mosquitoes. One-way ANOVA followed by Tukey’s multiple comparison tests were applied to test the differences in virus titer among samples but no significant differences in titers were found

To investigate the role of *Aa*FAS1 in DENV2 replication in vivo, mosquitoes were IT injected with dsRNA derived from *Aa*FAS1 or GFP genes and subsequently exposed to DENV2 infectious blood meal 2 days post-injection (Fig. 8). On days 0, 3 and 7 pbm (corresponding to days 2, 5 and 9 post-dsRNA injection), whole mosquitoes were collected and analyzed for *Aa*FAS1 gene expression (Fig. 8A). On the day of DENV2 infection by blood meal (2 days post-dsRNA injection), the level of *Aa*FAS1 expression was downregulated to 52.36 ± 26.51% relative to GFP-KD group. On day 3 pbm, *Aa*FAS1 expression recovered to 83.52 ± 45.13% and was comparable to the *Aa*FAS1 expression level in the GFP-KD control collected on the same day. On day 7 pbm, *Aa*FAS1 was upregulated to 189.29 ± 44.38%, suggesting a possible over-compensation post KD effect (Fig. 8A).

Investigation of DENV2-fed mosquitoes showed that, using pairwise *χ*^2^ tests, the percent of infection in the GFP-KD group (negative KD control) was higher compared to the percent infection of DENV2-KD groups (positive KD control) at all time points (Fig. 8B–D and Additional file 1: Table S6), indicating that the negative and positive dsRNA KD controls were effective against DENV2 infection in mosquitoes. Interestingly, we observed significant differences of percent infection between *Aa*FAS1-KD and GFP-KD in midgut samples on day 3 pbm, suggesting that *Aa*FAS1-KD may have a detrimental effect on virus infection in the mosquito midgut (Fig. 8B). However, the inhibitory effect of *Aa*FAS1-KD on DENV2 infection did not persist beyond day 3 pbm since the differences in percent infection between *Aa*FAS1-KD and GFP-KD mosquitoes on days 7 and 14 pbm were not observed (Fig. 8C, D. Additional file 1: Table S6).

Among the mosquitoes that were infected with DENV2 viruses, we did not observe significant differences in viral titers in any treatment at any time point (Fig. 8B–D, Additional file 1: Table S7). However, we observed two distinct populations of viral titers in *Aa*FAS1-KD carcasses on days 14 pbm (Fig. 8D, Additional file 1: Table S7); some with viral titers comparable to GFP-KD control (5.8 × 10^4^ PFU/carcass (i) and some with distinctively lower titers (3 PFU/carcass (ii) (Additional file 1: Table S7). This observation suggests that transient KD of *Aa*FAS1 may have a prolonged effect that impacts dissemination of the virus in mosquitoes.

## Discussion

Lipids are essential for a variety of physiological processes in mosquitoes [3, 10, 12, 41, 42]. Mosquitoes not only acquire lipids from maternal (i.e., deposition to eggs) and dietary sources, but they also have the ability to synthesize lipids de novo. In this study, we characterized the expression of the *Aa*FAS gene family, key enzymes in the de novo lipid biosynthesis pathway. Additionally, we investigated the potential role of *Aa*FAS1 in supporting DENV2 replication in the mosquito cell line and the mosquito vector.

In this study, we identified seven putative *Aa*FAS genes (*Aa*FAS1-6 and *Aa*FAS-like) in the AaegL5 assembly based on amino acid similarity to FAS from vertebrates, invertebrates and yeast. Amino acid sequence alignments and domain analyses revealed low amino acid similarity between mosquito and human FAS (< 50%), including the absence of the ΨME domain. This was also observed in other insect FAS, such as *D. melanogaster* (fruit fly), *Bombyx mori* (silkworm), *A. mellifera* (honeybee), *Culex pipiens* and *An. gambiae.* While the ΨME domain in human FAS is still present, it lacks the conserve sequence motif for S-adenosyl-methionine (SAM)-dependent methyltransferases, which is highly conserved in bacteria and fungi, resulting in an absence of methyltransferase activity [1]. This may reflect that this domain is unnecessary for metazoan FAS.

Gene duplication is a hallmark of many mosquito gene families and has been proposed as a source of new evolutionary features [23, 38, 43]. Retention of duplicated genes may be indicative of positive/neutral selection and loci associated with a fitness advantage for the mosquito [44]. We performed molecular and preliminary functional characterization of the *Aa*FAS gene family and detected transcripts for five of the seven *Aa*FAS genes. However, we were unable to detect transcripts for either *Aa*FAS6 or *Aa*FAS-like, suggesting that these gene models likely represent pseudogenes or may reflect an issue in the assembly.

Since mosquitoes undergo four distinct developmental stages in their life and these stages possess very distinct habitats and food sources, different *Aa*FAS genes may play roles supporting the unique requirements for FAS in these different life stages. Transcriptional profiles of *Aa*FAS1-5 revealed low expression levels for all *Aa*FAS in larval and pupal stages, suggesting that these genes may not be constitutively active across the mosquito life cycle. We speculated that maternal lipid deposition in eggs during oogenesis (these comprise about 35% of dry egg weight [7]) and larvae diets may serve to support the metabolic needs during these stages [8, 9, 45]. Thus, they may have minimal requirement for de novo fatty acid biosynthesis. Moreover, in male mosquitoes, we observed high levels of expression of all *Aa*FAS, except *Aa*FAS4. Male *Ae. aegypti* do not blood feed, but solely obtain their diet from plant nectar, honeydew and fruits [46]. Since these diets are high in carbohydrate but low in lipid content, high expression of *Aa*FAS genes in male mosquitoes may reflect a dependency on *Aa*FAS for de novo synthesis of lipids.

While validation of *Aa*FAS genes and their role in de novo fatty acid synthesis is beyond the scope of this study, a previous study by Alabaster et al. [12] showed that transient KD of *Aa*FAS1 and the rate limiting enzyme for fatty acid synthesis, acetyl-coA carboxylase, caused lower numbers of egg deposition and nonviable eggs, respectively. Therefore, this study validated the roles of these enzymes and their importance in mosquito physiology.

In this study, expression analyses also revealed that *Aa*FAS1 is the predominant *Aa*FAS transcript in both male and female mosquitoes. It has the highest amino acid similarity to the human and mouse FAS. Upon *Aa*FAS1-KD in female mosquitoes, we observed a two-fold increase in other *Aa*FAS transcripts, indicating an attempt to compensate for the loss of *Aa*FAS1 expression (Fig. 6A). The expression of these genes may have failed to compensate for the loss of *Aa*FAS1, since the expression levels of these *Aa*FAS transcripts were still lower than the remaining *Aa*FAS1 expression post-KD. Improving the KD efficiency or extending the period of KD of *Aa*FAS1 (such as using CRISPR/Cas9 knockout) may provide further insights into the redundancy of these *Aa*FAS genes.

Since previous studies have demonstrated the importance of FAS activity in flavivirus replication in both human and mosquito cells [13, 15, 16], we wanted to investigate whether *Aa*FAS1 also played an important role in DENV2 infection in the mosquito vector. Indeed, KD of *Aa*FAS1 showed significant inhibition of DENV2 infection in both *Ae. aegypti* cells in culture and mosquito midguts. However, the inhibitory effect of virus infection in the midgut was only observed on day 3 pbm, as seen by the reduced percent infection in *Aa*FAS1-KD mosquitoes compared to the GFP-KD group. This phenomenon might be caused by the transient KD of *Aa*FAS1 transcripts. Further studies with longer suppression of *Aa*FAS1 expression would be required to demonstrate the prominent impact of *Aa*FAS1 on infection and transmission.

Interestingly, we found an upregulation of *Aa*FAS1 expression (~ 200% increase compared to the *Aa*FAS1 levels in the GFP-KD control) on day 9 post-KD. Further studies are needed to better understand the biological impact of this “rebound” effect as it may have relevance for strategies aimed at suppression of host factors to disrupt pathogen transmission. In this study, we did not observe increased virus infection in these ‘rebound’ mosquitoes above the GFP-KD levels, suggesting that elevated *Aa*FAS1 transcripts may not necessarily equate to increased *Aa*FAS1 activity. Alternately, the ability of *Aa*FAS1 to support DENV2 replication in mosquitoes might have reached its limits.

Additionally, though the expression of *Aa*FAS1 was knocked down only transiently, we observed a prolonged effect on virus transmission. We found a separation of the virus titers into two groups—high (Fig. 8D, i) and low (Fig. 8D, ii)—in the carcass of the *Aa*FAS1-KD mosquitoes. A study by Ye et al. [47], showed that mosquitoes that were IT injected with DENV at 10^6^ PFU expectorated DENV into the saliva at about 10^2^ PFU, while mosquitoes that were IT injected with DENV at 10^7^ PFU expectorated DENV into the saliva at about 10^4^ PFU. These results suggests that viral titer of DENV in saliva may be dependent on the titer in the body (disseminated titers). In our observations, it is possible that mosquitoes with low body titers (group ii) may not be able to transmit the virus efficiently and the KD of *Aa*FAS1 may result in a reduction of transmission potential. This study suggests that biological relevance of low viral titers in carcasses and its impact on transmission dynamics are worthy of further investigation.

## Conclusion

Here we present expression analyses of the *Aa*FAS gene family and a focused study of *Aa*FAS1 during DENV2 infection in *Ae. aegypti*. We annotated seven *Aa*FAS genes from the AaegL5 genome assembly and present evidence to support the function of five genes. Expression data revealed complexities of *Aa*FAS expression between stages and sexes and suggest that *Aa*FAS1 is the predominant transcript in both male and female adult mosquitoes. Sequence homology suggested conservation between mammalian FAS and *Aa*FAS1, and the presence of multiple catalytic domains supports *Aa*FAS1 as a predominant enzyme in the *Aa*FAS family in de novo lipid biosynthesis in *Ae. aegypti*. In addition, *Aa*FAS1 was found to facilitate DENV2 replication in both cell culture and in *Ae. aegypti*. In the latter case, it demonstrated the potential to affect vector competency for virus transmission.

## Supplementary Information

**Additional file 1: Figure S1.** Alignment of the conserved YKELRLRGY motif. **Figure S2.** Amino acid alignment of the pseudo-methyltransferase (ΨME) domain of *H. sapiens* and *Ae. aegypti* FAS. **Figure S3.** RT-PCR assays designed to detect mRNA products of *Aa*FAS-like and *Aa*FAS6. **Table S1.** List of vertebrate, invertebrate and yeast FAS gene models employed in the present study. **Table S2.** Primers for generation of dsRNA for knock-down studies. **Table S3.** Primers for *Aa*FAS expression analyses. **Table S4.** Primers for RT-PCR assay detecting mRNA products *Aa*FAS-like and *Aa*FAS6. **Table S5.** Amino acid similarity of FAS domains between *H. sapiens* and *Ae. aegypti.* **Table S6.** Comparisons of percent infection in mosquitoes (*P*-values). **Table S7.** Virus titers in mosquito tissues.

**Additional file 2: File S1.** mRNA and amino acid sequences of 7 *Aa*FAS genes.

## Data Availability

All data and materials were presented in the manuscript and Additional file.

## Abbreviations

ACC: Acetyl-CoA carboxylase
*Ae. aegypti*: *Aedes aegypti*
*Aa*FAS: *Aedes aegypti* fatty acid synthase
bp: Base pair
cDNA: Complementary deoxyribonucleic acid
DENV2: Dengue virus serotype 2
dpi: Days post-infection
dsRNA: Long double-stranded ribonucleic acid
FAS: Fatty acid synthase
FBS: Fetal bovine serum
GFP: Green fluorescent protein
IT: Intrathoracic
KD: Knockdown
MEM: Minimum essential media
PBS: Phosphate-buffered saline
mRNA: Messenger ribonucleic acid
PCR: Polymerase chain reaction
qPCR: Quantitative polymerase chain reaction
pbm: Post-blood meal
PFU: Plaque forming unit
ΨME: Pseudo-methyltransferase
RNA: Ribonucleic acid
RNAi: Interference ribonucleic acid
RT: Reverse transcription
RT-PCR: Reverse transcription polymerase chain reaction
SNP: Single nucleotide polymorphism
